# *QuickStats:* Age-Adjusted Death Rates* from Diabetes Mellitus^†^ as Underlying or Contributing Cause Among Adults Aged ≥65 Years, by Race/Ethnicity — National Vital Statistics System, United States, 2004–2017

**DOI:** 10.15585/mmwr.mm6824a6

**Published:** 2019-06-21

**Figure:**
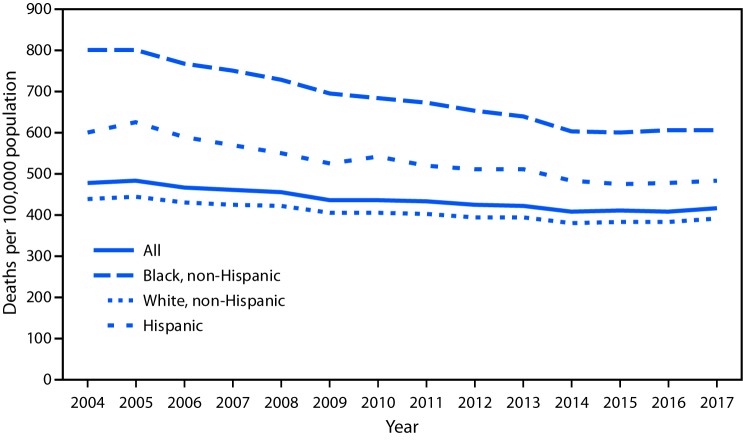
During 2004–2017, the death rate from diabetes mellitus as underlying or contributing cause among adults aged ≥65 years decreased from 477.5 per 100,000 in 2004 to 418.1 in 2017. Throughout this period, the death rate was highest among non-Hispanic black adults and lowest among non-Hispanic white adults. During 2004–2017, the death rate decreased from 438.3 per 100,000 to 391.1 among non-Hispanic white adults, from 602.0 to 485.7 among Hispanic adults, and from 804.3 to 607.0 among non-Hispanic black adults.

**Source:** National Vital Statistics System, 2004–2017. https://www.cdc.gov/nchs/nvss/deaths.htm.

